# Whole exome sequencing approach to childhood onset familial erythrodermic psoriasis unravels a novel mutation of CARD14 requiring unusual high doses of ustekinumab

**DOI:** 10.1186/s12969-019-0336-3

**Published:** 2019-07-08

**Authors:** S. Signa, E. Campione, M. Rusmini, S. Chiesa, A. Grossi, A. Omenetti, R. Caorsi, G. M. Viglizzo, M. Galluzzo, L. Bianchi, M. Talamonti, A. Orlandi, A. Martini, I. Ceccherini, M. Gattorno

**Affiliations:** 1 Centro Malattie Autoinfiammatorie e Immunodeficienze- Clinica Pediatrica e Reumatologia, IRCCS Giannina Gaslini, Via Gaslini 5, 16147 Genova, Italy; 2 Dipartimento di Neuroscienze, Riabilitazione, Oftalmologia, Genetica e Scienze Materno-Infantili (DINOGMI), Università di Genova, Genoa, Italy; 3 Dermatology, Department of “Medicina dei Sistemi”, University of Rome Tor Vergata, Rome, Italy; 4 UO Genetica Medica, IRCCS G. Gaslini, Genoa, Italy; 5 UO Dermatologia , IRCCS G. Gaslini, Genoa, Italy; 6 Cattedra di Anatomia Patologica, Policlinico Universitario di Roma “Tor Vergata”, Rome, Italy; 7 Direzione Scientifica, IRCCS G. Gaslini, Genoa, Italy

**Keywords:** Erythrodermic psoriasis, CARD14, Ustekinumab, Whole exome sequencing

## Abstract

**Background:**

Autosomal dominant gain of function mutations in caspase recruitment domain family member 14 (CARD14) is a rare condition associated with plaque-type psoriasis, generalized pustular psoriasis, palmoplantar pustular psoriasis and pityriasis rubra pilaris. Recently, a new CARD14 –associated phenotype defined as CAPE (CARD14-associated papulosquamous eruption) with clinical features of both psoriasis and pityriasis rubra pilaris was reported. We describe a family carrying a novel heterozygous mutation in CARD14 gene, with childhood-onset erythrodermic psoriasis requiring an unusual extremely high dose (up to 2 mg/kg every 8 weeks) of ustekinumab to achieve disease remission.

**Case presentation:**

We describe a large family with three pairs of twins presenting a clinical phenotype characterized by childhood-onset erythrodermic psoriasis; in some family members is also reported psoriatic arthritis. The two probands presented poor clinical response to topic and systemic therapy with antihistamine, steroid, retinoids, cyclosporine and etanercept. After exclusion of the most common genes associated to autoinflammatory diseases (*IL36RN, IL1RN, MVK, TNFRSF1A, NLRP3, NLRP12, MEFV, NOD2, PSMB8, PSTPIP1, LPIN2*) we approached a new gene search by subjecting to Whole Exome Sequencing (WES) analysis five members of the family. A novel heterozygous mutation (c.446 T > G, leading to the missense amino acid substitution p.L149R) in the exon 4 of the CARD14 gene was identified in all affected members. Increasing dosages (up to 2 mg/kg every 8 weeks) of ustekinumab, a human monoclonal antibody targeting interleukin-12 (IL-12) and interleukin-23 (IL-23), allowed the complete control of the clinical manifestations, with an evident reduction of circulating Th17 and Th22 CD4+ T cell subsets.

**Conclusions:**

We describe the association of mutations of the *CARD14* gene with an erythrodermic psoriasis pedigree, underlying the necessity to investigate CARD14 mutations in childhood-onset psoriasis cases and confirming the presence of CARD14 causative mutations also in erythrodermic psoriasis form, as recently reported. Also in pediatric age, ustekinumab represents a powerful therapeutic option for this rare condition, that is usually refractory to other treatments. In young children, high and frequent dosages allowed a complete control of the clinical manifestations without any severe side effects, with a long-term follow-up.

## Background

Gain of function dominant mutations in caspase recruitment domain family member 14 (CARD14) were found to cause plaque psoriasis in two families (30% of the members also developed psoriatic arthritis) and a monogenic form of severe generalized pustular psoriasis (CARD14-mediated psoriasis -CAMPS) [1]. CARD14 mutations have also been associated with pityriasis rubra pilaris (PRP) [2]. Variants of CARD14 can also be found in palmoplantar pustular psoriasis [3] and Psoriasis Vulgaris [4]. Recently, Craiglow et al. described a CARD14 –associated phenotype defined as CAPE (CARD14-associated papulosquamous eruption) with clinical features of both psoriasis and PRP [5]. Finally, the wide spectrum of CARD14-mediated clinical presentations has been included in the new concept of CARD14-related autoinflammatory keratinization diseases (CAIKDs) [6].

The therapeutic approach in CAIKDs includes drugs used for the treatment of moderate-to-severe psoriasis, i.e. methotrexate, cyclosporine, and biological agents such as anti-TNF agents [7]. Ustekinumab is a fully human IgG1_k_ monoclonal antibody that notably targets the common p40 subunit of IL-12 and IL-23, well-defined key mediators of psoriasis that are able to stimulate two emerging Th-cell subsets of CD4 + T, Th17 and Th22 cells with a crucial role in immune response to tissue inflammation. It has been reported to be effective in moderate-to-severe psoriasis and is actually indicated for the treatment of moderate to severe plaque psoriasis in adults and adolescent patients from the age of 12 years and older, who are inadequately controlled by, or are intolerant to other systemic therapies or phototherapies [8]. It is also indicated for the treatment of active psoriatic arthritis in adult patients, when the response to previous non-biological disease-modifying anti-rheumatic drug (DMARD) therapy has been inadequate. The use of ustekinumab in patients younger than 12 years old is reported only anecdotally [9], whereas its use in CAIKDs patients is reported but the administration schedule often needs adjustments compared with the traditional one [5, 10, 11].

## Case presentation

**Fig. 1:**
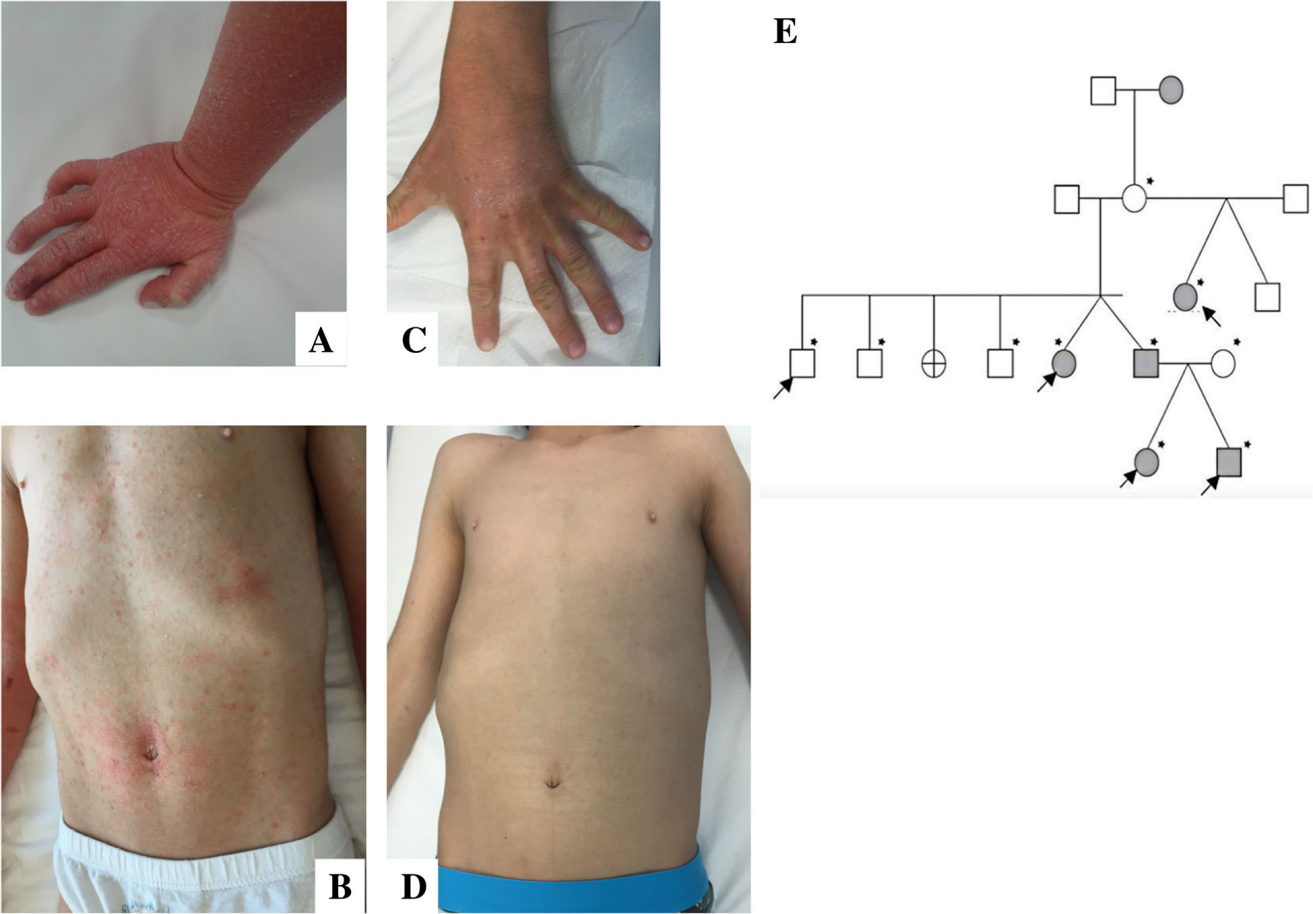
**a-d** Clinical presentation of the two twins presenting a CARD14-mediated severe erythrodermic psoriasis; hand (**a**) and trunk (**b**) before treatment and after (**c**-**d**) seven months of ustekinumab treatment. **e** The whole pedigree is depicted with the grey symbols indicating the affected members and an asterisk pointing out members for whom we got a biological sample. Five members of the family, namely those indicated by an arrow were analyzed for a new gene search by Whole Exome Sequencing (WES) analysis. The two twin siblings of the IV generation are the probands

**Fig. 2:**
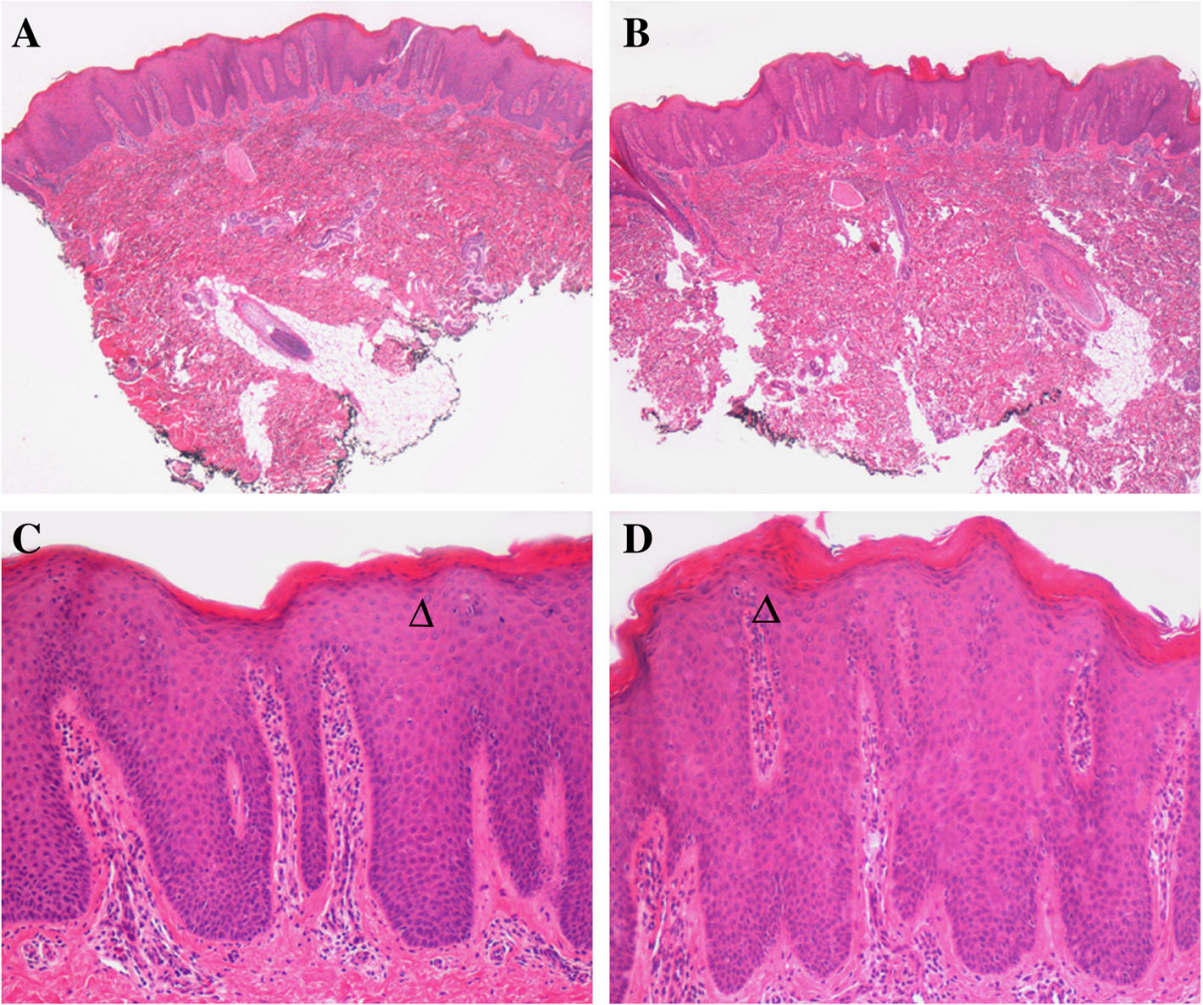
Microscopic evaluation of twin skin biopsies. At different magnification, microscopic evaluation of Haematoxylin&Eosin-stained paraffin sections of skin biopsies of both twins (**a**-**c** and **b-d**, respectively) reveals a parakeratotic cornified layer and epidermis with marked elongation of rete ridges and an almost absent granular layer (arrow heads); in the papillary dermis, inflammatory cells surround dilated tortuous small vessels, consistent with the diagnosis of psoriasis. Haematoxylin&Eosin stain; original magnification **a**, **b**: 40X; **c**, **d**: 100X

Two 7 year-old dizygotic twins with an history of severe erythrodermic dermatitis were evaluated for a possible monogenic autoinflammatory disease. Both twins showed an itching erythematous desquamative dermatitis involving the face, the limbs and the trunk (Fig. 1a-b). Hyperkeratosis was prominent at the tip of nose, elbows, knees, and dorsal surface of the hands. Onychodistrophy of the fingers was also present in the male patient. The two children were already clinically described [12]. Since the age of 9 months, both children presented with unmanageable erythroderma, with severe itching, onychodystrophies and fissures in both children, and ectropion in the sister. No “islands of sparing”, typical of PRP, were evident. Low-grade fever, mild leukocytosis and elevation of ESR were occasionally observed in association with the severe skin flares. Microscopic evaluation of Haematoxylin&Eosin-stained paraffin sections of skin biopsies of both twins revealed a parakeratotic cornified layer and epidermis with marked elongation of rete ridges and an almost absent granular layer. In the slight oedematous papillary dermis, inflammatory cells surround dilated tortuous small vessels; these microscopic findings were consistent with the diagnosis of psoriasis (Fig. 2).

Since disease onset both children were treated with local and systemic steroids with a good response at high doses but several relapses at steroid tapering. They also received a short cycle of cyclosporine at low-dosage for one month followed by etanercept (0.8 mg/kg), without evident results. Cyclosporine at a higher dosage of 5 mg/kg/day was therefore started. After three months of treatment, only a partial clinical improvement was achieved, despite the high dosage. Combination therapy with retinoids was also attempted for 1 month, without any substantial additional improvement.

The analysis of the family was definitely evocative for an autosomal dominant skin disease (Fig. 1e). Five out of six twins, belonging to three pairs of dizygotic twins present along the four generation family pedigree, were affected by plaque psoriasis and two of them presented with psoriatic arthritis. The father of the two probands displayed the same cutaneous picture.

In the first step, we analyzed the most common genes associated with autoinflammatory diseases through a next generation sequencing (NGS) based diagnostic panel including the following genes: *IL1RN, MVK, TNFRSF1A, NLRP3, NLRP12, MEFV, NOD2, PSMB8, PSTPIP1, LPIN2* [13]. Mutations of the IL36RN gene were excluded by Sanger Sequencing. Whole Exome Sequencing (WES) analysis were therefore performed on five members of the family, indicated by an arrow in Fig. 1e. Samples were subjected to Whole Exome Sequencing (WES) in outsourcing, and raw data were transferred to our lab for the bio-informatic analysis. FastQ raw data were analyzed by FastQC software to check the quality of the run in terms of length and GC content of reads, quality of nucleotides within the reads, over-represented sequences (PCR duplicates or excess of adaptors), etc. Alignment of the sequences against the Hg19 genome reference was followed by variant calling, assessed by the Haplotype Caller tool of the newest version of GATK.

Among variants shared by the four affected individuals and not present in the unaffected paternal uncle, a missense mutation of the CARD14 gene resulted worth of further investigation. In particular, it was the case of an exon 4 heterozygous nucleotide change, c.446 T > G, leading to the novel missense amino acid substitution p.Leu149Arg (p.L149R). The presence of this variant was validated by Sanger sequencing in the affected members who underwent WES, and in the rest of the available family members. This allowed to confirm the segregation of the CARD14 mutation with the disease phenotype.

After the identification of the molecular defect, treatment with ustekinumab was started at the dosage of 0.75 mg/kg at week 0, week 4 and subsequently every 12 weeks. Ustekinumab is a fully human IgG1_k_ monoclonal antibody that notably targets the common p40 subunit of IL-12 and IL-23, well-defined key mediators of psoriasis that are able to stimulate two emerging novel CD4 (+) Th-cell subsets, Th17 and Th22, with a crucial role in immune response to tissue inflammation. At week 4 and week 16 both twins showed a substantial improvement of their clinical conditions (PASI: value baseline 52, and declined to 5.3 after treatment at week 16), with a good tolerance and no side effects. Between the first and second administration, the cyclosporine dosage was reduced of 50%. Despite the good control of the disease, both children displayed the tendency to minor flares after 2 months from the last administration. For this reason, a new schedule for ustekinumab treatment was used, with administrations every 8 weeks. At the same time, cyclosporine was definitively withdrawn. At the evaluation at week 24 the boy showed an almost complete resolution of dermatological manifestations. Conversely, the sister presented a mild disease flare, likely due to an undercurrent chicken-pox infection that resolved regularly and without complications. Aiming the complete clinical remission, the dose of ustekinumab was subsequently increased to 2 mg/kg every 8 weeks. At week 28 both patients showed a complete resolution of skin lesions (Fig. 1c-d). Neither further relapses nor side effects were subsequently developed during follow-up. After 26 months of treatment the patients were free of any clinical manifestation with a PASI ranging from 4 to 6, and no adverse events have been manifested so far. The father of the two children was previously treated with cyclosporine, and subsequently also with different anti-TNF agents (etanercept, infliximab and adalimumab) with poor results. He also started ustekinumab at the dose of 90 mg every 12 weeks with a partial resolution of skin manifestations, but resolution of itching. The other affected family members were not available for the direct clinical evaluation in our centers.

In order to evaluate the impact of ustekinumab treatment on the frequencies of Th17 and Th22 cells, we decide to perform an analysis of IFN-γ, IL-17 and IL-22 producing cells at baseline and after ustekinumab treatment. Blood samples were collected at baseline, at week 4 and at week 24 post-ustekinumab treatment. Peripheral blood mononuclear cells (PBMC) were isolated from patients and stimulated with PMA/ionomycin and Brefeldin A for 6 h at 37 °C. Briefly, for analyzing cytokine production from T cells, cells were firstly stained with Percp-Cy™ 5.5 anti-human CD4 monoclonal antibody (MAb) (BD Pharmingen™). After treatment with fixation and permeabilization wash buffer, cells were incubated with Fitc anti-human IFN-γ (BD Biosciences), Alexa Fluor 647 anti-human IL-17A (e-Bioscience), and Phycoerythrin (PE) anti-human IL-22 (e-Bioscience) MAbs. Fluorescence profiles were assessed by flow cytometry using FACS Canto II (Becton Dickinson) and the data were analyzed using FlowJo software (LLC).

**Fig. 3:**
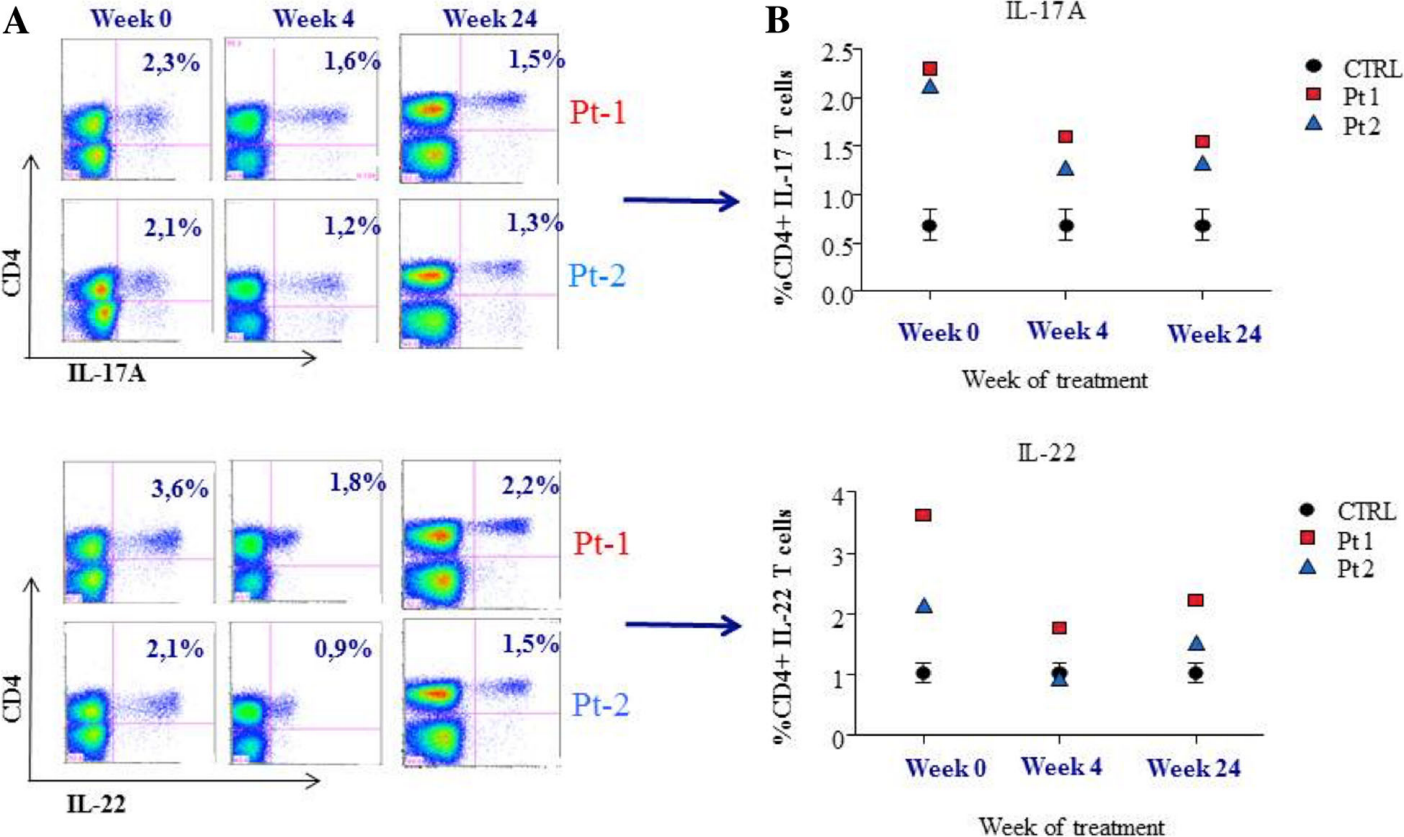
Percentange of circulating Th17 (IL-17A) and Th22 (IL-22) in stimulated PBMCs of CAMPs patients before and after ustekinumab treatment. **a** On the left, representative flow cytometry dot plots of the intracellular expression of IL-17 and IL-22 from CAMPS patients (Pt) (at baseline, at week 4 and at week 24); (**b**) On the right: the change in circulating frequency of Th17 and Th22 cells in CAMPS patients and pediatric healthy controls (CTRLs) before and after ustekinumab treatment (at baseline, at week 4, at week 16). Each point represents a sample

At baseline, the frequency of Th17 and Th22 cells was higher in CAMPS children when compared to the controls (IL-17A: Pt1 2,3%, Pt2: 2,1% versus CTRL mean: 0.8%; IL-22: Pt1 3,6, Pt2: 2,15 versus CTRL (control) mean value: 1,1) (Fig. 3, panels A, B). Conversely, IFN-gamma producing Th-1 cells did not differ between patients and controls (data not shown). After PBMCs stimulation, the levels of IL-17A and IL-22 producing cells at week 4 and 24 decreased after ustekinumab treatment, consistent with the clinical findings. As shown in the dot plots Fig. 3 (panels A, B) and in related graphs, both circulating Th17 and Th22 were reduced at week 4 (IL-17: Pt1 1.6%, Pt2 1.2% versus CTRL mean value 0.8%; IL-22: Pt1 1.8%, Pt2 0.9% versus CTRL mean value: 1,1) and at week 16 (IL-17: Pt1 1.5%, Pt2 1.3% versus CTRL mean value: 0.8%; IL-22: Pt1 2.2%, Pt2: 1.5% versus CTRL mean value: 1,1%).

## Discussion and conclusions

Mutations of the CARD14 gene have been described as associated with PRP and several psoriasis clinical phenotypes. Whereas adult onset CARD14-related disease mainly displays plaque psoriasis and palmoplantar pustular psoriasis, childhood onset phenotype is often associated with more severe clinical manifestations such as generalized pustular psoriasis [1–4, 14, 15]. More recently, Craiglow et al [5] described a spectrum of CARD-14 related disease named CAPE (CARD14- associated papulosquamous eruption) which presents features of both psoriasis and PRP and a classical childhood onset. Among the 15 patients listed, two of them are described as erythrodermic.

In our report, we describe the association of mutations of the *CARD14* gene with an erythrodermic psoriasis pedigree. The therapeutic approach in CAIKDs includes drugs used for the treatment of moderate-to-severe psoriasis [7] but, as observed in the present family, treatment with conventional therapies is often associated with a partial or incomplete response. The use of ustekinumab in patients younger than 12 years old is reported only anecdotally [9]. A good response to the monoclonal antibody has recently been described in adult patients with pityriasis rubra pilaris due to mutation of CARD14 [10, 11]. Craiglow et al. described 15 patients with CAPE and six of them were treated with ustekinumab with different administration schedules, even if it is not clear if they were treated with ustekinumab during childhood or adulthood. The schedules described a range from 0.7 mg/kg to 1.1 mg/kg every 12 weeks; in one severe case a dosage of 1.2 mg/kg every 8 weeks was required to achieve clinical improvement and the patient was eventually transitioned to guselkumab. So far, no data is available on the optimal schedule of ustekinumab administration (dose and frequency) in young children and on its efficacy and safety in the long run. In our case only an extremely high dose of the drug (2 mg/kg) every 8 weeks was able to completely control the skin manifestations, with no severe complication, namely higher rate of infections. The same high doses were also used in our recent experience with 2 children affected by the deficiency of IL-36 receptor antagonist (DITRA), with the same complete response and good safety profile [16]. The positive response to treatment with ustekinumab in both twins highlights the efficacy of this biological therapy in childhood onset erythrodermic psoriasis related to CARD14 mutations, even if a dose until four times the standard can be required to maintain stable clinical remission.

In conclusion, CARD14 gain of function mutations can give rise to unusual clinical phenotype characterized by diffuse erythrodermic psoriasis and mutations in CARD14 should always be considered in severe childhood onset psoriasis. Also in pediatric age, ustekinumab represents a powerful therapeutic option for this rare condition, that is usually refractory to other treatments. In young children, high and frequent dosages allowed a complete control of the clinical manifestations without any severe side effect, with a long-term follow-up.

## Data Availability

The datasets used and/or analysed during the current study are available from the corresponding author on reasonable request.

## Abbreviations

CAIKDs: CARD14-related autoinflammatory keratinization diseases
CAMPS: CARD14-mediated psoriasis
CAPE: CARD14-associated papulosquamous eruption
CARD14: Caspase recruitment domain family member14
DMARD: Disease-modifying anti-rheumatic drug
PASI: Psoriasis area severity index
PBMC: Peripheral blood mononuclear cells
PMA: Phorbolmyristate acetate
PRP: Pityriasis rubra pilaris
WES: Whole exome sequencing
